# Developing a risk prediction model for keratinocyte carcinoma in patients with actinic keratosis

**DOI:** 10.1111/bjd.18966

**Published:** 2020-05-10

**Authors:** Y. Kim, E. Jorgenson, M.M. Asgari

**Affiliations:** 1 Department of Population Medicine, Harvard Medical School and Harvard Pilgrim Health Care Institute, Boston, MA, USA;; 2 Massachusetts General Hospital, Department of Dermatology, Boston, MA, USA;; 3 Kaiser Permanente Northern California, Division of Research, Oakland, CA, USA

Actinic keratoses (AKs) commonly arise on skin exposed to chronic ultraviolet radiation. AKs have been associated with an increased risk of keratinocyte carcinoma (KC), including basal cell carcinoma (BCC) and squamous cell carcinoma (SCC).^1^ While the majority of AKs regress,^2^ up to 16% may undergo malignant transformation.^3^ Thus, it is important to identify a subset of patients with AK who are at increased risk for developing KC. There are several models for predicting KC based on known risk factors, including age, sex, pigmentation, sun sensitivity, freckling and history of skin cancer.^4,5^ Risk models incorporating genetic risk using polygenic risk scores or the number of risk alleles in KC development showed improved discriminative ability.^6,7^ Despite the increased risk for developing KC in patients with AK, a risk prediction model for KC that is developed among those with AK lesions has been lacking.

In this issue of the *BJD*, Tokez et al. propose a novel prediction model for incident KC among patients with AK using the Rotterdam Study cohort.^8^ They assessed potentially relevant phenotypic, lifestyle and genetic factors to produce their model, which demonstrates modest discriminative ability (*C*-statistic 0·60).^8^ Predictors in their final model included the number of AKs, anatomical location of AKs and coffee consumption. While the majority of AKs arise on chronically sun-exposed areas including the head/neck and upper extremities, in the proposed prediction model, AK lesions located on anatomical sites other than the head/neck or upper extremities were associated with a higher risk of developing KC. The authors reported that coffee consumption reduced KC risk; however, the level of support is limited, as indicated by the confidence interval in the multivariable analyses. Observational studies on the association between coffee intake and KC risk have shown conflicting results, although two recent meta-analyses reported a protective effect against BCC development.^9,10^ Future studies are needed to replicate the effect of coffee consumption in patients with AK.

The authors investigated the impact of a genetic risk score generated from seven BCC- and cutaneous SCC-associated single-nucleotide polymorphisms (SNPs) in their model selection: *BNC2/CNTLN, TYR, IRF4, HERC2, MC1R, SLC45A2* and *RALY/ASIP*. However, this score ultimately did not meet the criteria for inclusion in the final model. Three of these SNPs (*TYR, IRF4* and *MC1R*) were also associated with AK risk in a previous genome-wide association study, and perhaps the choice of SNPs for the genetic risk score might have impacted its lack of inclusion in the final model.^11^ Future genetic studies are warranted to improve the model’s discriminative ability.

Although these findings need to be externally validated, this work paves the way for a prediction of KCs developing in patients with AK by providing a risk prediction model and a tool to quantify the combined contribution of the risk factors. This tool integrated readily accessible factors and can be used to aid identification of patients in need of increased skin cancer prevention efforts and dermatological surveillance. Future studies may help validate and optimize this risk prediction model and tool.
